# Protective Effects of Sesamin against UVB-Induced Skin Inflammation and Photodamage In Vitro and In Vivo

**DOI:** 10.3390/biom9090479

**Published:** 2019-09-12

**Authors:** Tzu-Yu Lin, Po-Yuan Wu, Chien-Wei Hou, Ting-Yi Chien, Qiao-Xin Chang, Kuo-Ching Wen, Chien-Yih Lin, Hsiu-Mei Chiang

**Affiliations:** 1 Department of Cosmeceutics, China Medical University, Taichung 40402, Taiwan; fishlin522@gmail.com (T.-Y.L.); pp85462@gmail.com (T.-Y.C.); nancystar597@gmail.com (Q.-X.C.); kcwen0520@mail.cmu.edu.tw (K.-C.W.); 2 Department of Dermatology, China Medical University Hospital, Taichung 40402, Taiwan; wu.poyuan@gmail.com; 3 School of Medicine, China Medical University, Taichung 40402, Taiwan; 4 Department of Biotechnology and Pharmaceutical Technology, Yuanpei University of Medical Technology, Hsinchu 30015, Taiwan; rolis.hou@mail.ypu.edu.tw; 5 Department of Biotechnology, Asia University, Taichung 41354, Taiwan; yihlin@asia.edu.tw; 6 Ph.D. Program for Biotechnology Industry, China Medical University, Taichung 40402, Taiwan

**Keywords:** sesamin, photodamage, inflammation, antioxidant, nuclear factor-kappa B, inhibitor κB

## Abstract

Ultraviolet (UV) exposure has been demonstrated as the most critical factor causing extrinsic skin aging and inflammation. This study explored the protective effects and mechanisms of sesamin against skin photodamage. Sesamin reduced intracellular reactive oxygen species production after UVB irradiation in human dermal fibroblasts. The sesamin treatment attenuated mitogen-activated protein (MAP) kinase phosphorylation and matrix metalloproteinase (MMPs) overexpression induced by UVB exposure, and it significantly enhanced the tissue inhibitor of metalloproteinase-1 protein expression. Sesamin also elevated the total collagen content in human fibroblasts by inhibiting UVB-induced mothers against decapentaplegic homolog 7 (Smad7) protein expression. Sesamin reduced UVB-induced inducible nitric oxide synthase (i-NOS) and cyclooxygenase-2 (COX-2) overexpression and inhibited nuclear factor-kappa B (NF-κB) translocation. Moreover, sesamin may regulate the c-Jun N-terminal kinases (JNK) and p38 MAP kinase pathways, which inhibit COX-2 expression. Sesamin could reduce UVB-induced inflammation, epidermal hyperplasia, collagen degradation, and wrinkle formation in hairless mice. It also reduced MMP-1, interleukin (IL-1), i-NOS, and NF-κB in the mouse skin. These results demonstrate that sesamin had antiphotodamage and anti-inflammatory activities. Sesamin has potential for use as a skin protection agent in antiphotodamage and skin care products.

## 1. Introduction

Skin is the external part of the human body and plays a crucial role in protection from the exogenous invasion of pathogenic factors or pollutants. Long-term exposure to ultraviolet (UV) irradiation induces visible signs of aging, known as photoaging, which include characteristics such as wrinkles, pigmentation, sagging, and inflammation. Exposure to solar light induces oxidative stress and inflammation in the skin, resulting in the activation of aging-related pathways and causing skin aging and damage [1]. The extracellular matrix (ECM) is abundant in the dermis and is responsible for maintaining the skin structure and resilience. The major components of the ECM are the Type I and III collagen, along with small amounts of elastin and glycoproteins [2]. Matrix metalloproteinases (MMPs) are responsible for the degradation of the ECM, whereas tissue inhibitors of metalloproteinases (TIMPs) may inhibit the activity of MMPs [3]. In addition, many factors, such as inflammation and reactive oxygen species (ROS), may cause ECM degradation. Regulation of collagen gene expression is related to the physiology and pathology of skin aging and disorders [4].

UV exposure triggers two transcription factor pathways, namely the activator protein-1 (AP-1) and nuclear factor-kappa B (NF-κB) families, which modulate the expression of various UV responses [5]. Inhibitor κB (IκB) generally binds with NF-κB in the cytoplasm to create an inactive complex; however, UV exposure or ROS promotes IκB ubiquitination, triggering NF-κB translocation into the nucleus and causing inflammation [6]. NF-κB activation stimulates MMP-1 production, which degrades collagen in the dermis and results in wrinkle formation. UV irradiation upregulates various cytokines, such as interleukins (ILs), inducible nitric oxide synthase (i-NOS), and cyclooxygenase (COX)-2, and this consequently stimulates inflammation and results in skin sunburn and erythema. IL and NF-κB upregulate COX-2 expression, causing photodamage of the skin [7].

The seeds of *Sesamum indicum* Linn. (*Pedaliaceae*) have been used as a seasoning and cooking oil in the eastern part of Asia. They contain abundant lignans, such as sesamol, sesamin, and seamolin, which have been reported to exhibit an excellent antioxidative activity [8,9]. Sesamin oil was reported to promote wound healing and have antioxidative, anti-inflammatory, and hypolipemia activities [10,11,12]. Our previous study demonstrated that sesamol inhibited melanin synthesis in B16F10 cells and C57BL/6 mice [13,14]. Sesamin has also been reported to exhibit antioxidative, anti-inflammatory, and antinociceptive activities [15,16,17]. It scavenges ROS and nitric oxides (NO) and inhibits proinflammatory cytokines production, protecting the liver from injury in rats [18]. Sesamin elevated tocotrienol contents in the skin to reduce sunburn and the incidence of tumors [19]. We hypothesized that sesamin, with its potent antioxidative and anti-inflammatory activities, may exhibit benefits for the skin photodamage. The aim of the present study was to investigate the activity and mechanism of sesamin against photodamage and photoinflammation induced by UVB irradiation in human skin fibroblasts (Hs68 cells) and hairless mouse skin.

## 2. Materials and Methods

### 2.1. Materials

All reagents and chemicals used in the present study were the reagent grade. Reagents used in the cell culture, including Dulbecco’s modified Eagle’s medium (DMEM), fetal bovine serum (FBS), penicillin, and streptomycin, were obtained from Gibco, Invitrogen (Carlsbad, CA, USA). Albumin (from bovine serum), 2′,7′-dichlorofluorescin diacetate (DCFDA), dimethyl sulfoxide, leupeptin, paraformaldehyde, and phenylmethylsulfonyl fluoride were purchased from Sigma-Aldrich Corporation (St. Louis, MO, USA). Sodium dodecyl sulfate (SDS), Tris, and Tween 20 were obtained from the USB Corporation (Cleveland, OH, USA). The ECL western blotting detection reagent was obtained from Amersham Biosciences (Little Chalfont, England). MAP kinase inhibitors, including c-Jun N-terminal kinases (JNK) inhibitor II, PD98059, and SB203580, were obtained from Calbiochem (Darmstadt, Germany).

### 2.2. Cell Culture and UV Irradiation

Hs68 cells were purchased from the Bioresource Collection and Research Center (Hsinchu, Taiwan), seeded in DMEM with 10% FBS and 100 U/mL penicillin/streptomycin, and incubated at 37 °C with 5% CO_2_. A CL-1000M UV crosslinker with two UV lamps (peak wavelength at 302 nm) was applied for UV irradiation (UVP, Upland, CA, USA). Cells covered with PBS were exposed to 80 mJ/cm^2^ UVB for the DCFDA assay and 40 mJ/cm^2^ UVB for other experiments as described previously [20].

### 2.3. Detection of ROS Production

A DCFDA fluorescence assay was performed to study the intracellular ROS levels in Hs68 cells after UVB exposure as described previously [20]. Briefly, the cells were treated with various concentrations of sesamin after UVB irradiation. DCFDA was added to the cells after which the cells were incubated for 30 min. Fluorescence was detected at an emission wavelength of 520 nm and excitation wavelength of 488 nm using a microplate reader (Thermo Electron Corporation, Vantaa, Finland), and photos were captured using a fluorescence microscope (Leica DMIL, Wetzlar, Germany).

### 2.4. Western Blot Analysis

The protein expression of sesamin in Hs68 cells after UVB irradiation was assayed through western blotting [20]. Fibroblasts were harvested and lysed using the lysis buffer. The proteins were separated by gel electrophoresis on an SDS-polyacrylamide gel and recognized by specific antibodies. The proteins were probed with antibodies after transferring to polyvinylidene difluoride membranes. An ECL western blotting detection system (Fujifilm, LAS-4000, Tokyo, Japan) was used to detect the proteins, and the densities were measured using a densitometric program (MultiGauge V2.2, Fuji Pharma, Tokyo, Japan).

### 2.5. Measuring Total Collagen Synthesis in Fibroblasts

The total collagen synthesis in fibroblasts was detected using a Sircol collagen detection kit [21]. After UVB exposure and the sesamin treatment, the cell culture medium was used to measure the collagen content. Isolation and concentration reagents were mixed with the culture medium and incubated. Finally, the Sircol dye was added to the sample. After centrifugation, the washing reagent was mixed with the pellets, and the sample was centrifuged again. An alkali reagent was added to dissolve the precipitate, and the absorbance was detected at 555 nm.

### 2.6. Immunofluorescence Staining

Hs68 cells were seeded on coverslips and treated with UVB irradiation. Subsequently, sesamin at various concentrations was added to the cells after which the cells were incubated overnight. The cells were incubated with primary and Alexa Fluor 488 antirabbit IgG secondary antibodies (Invitrogen, Waltham, MA, USA). Finally, the cells on the coverslip were stained with the ProLong Gold antifade reagent (Thermo Fisher Scientific Inc, Waltham, MA, USA) and examined under a fluorescence microscope [22].

### 2.7. Effect of Sesamin Treatment on Photodamage in Hairless Mice

#### 2.7.1. Animals

Five-week-old female BALB/cAnN.Cg-Foxn1nu/CrlNarl mice were obtained from the National Laboratory Animal Center in Taipei, Taiwan. Animals were kept in the animal center of China Medical University and allowed to accommodate to the environment for one week. Protocols for animal experiments were approved (104-240-B) by the Institutional Animal Use and Care Committee of China Medical University.

#### 2.7.2. Experimental Design

The mice were randomly divided into the following five groups: Non-UVB irradiation or nonsesamin treatment (normal), UVB-irradiated, vehicle-treated and UVB-irradiated (vehicle), UVB-irradiated and 50-μM-sesamin-treated (UVB + 50 μM sesamin), and UVB-irradiated and 200-μM-sesamin-treated (UVB + 200 μM sesamin) groups. Hairless mice were exposed to gradient doses of UVB irradiation as described previously [20]. PEG400 (50 μL) was topically applied on the dorsal skin after UVB exposure in the vehicle-treated group daily, and 50 μL of 50 and 200 μM sesamin was applied to the appropriate sesamin groups. At the end of the experiment, the exposed areas were excised and then immersed in 10% formaldehyde in the PBS. The slides were mounted with a coverslip, stained in hematoxylin and eosin or Masson’s trichrome, and examined under a microscope or applied for immunohistological analysis as previously described [23].

#### 2.7.3. Detection of Erythema (a* Value) and Transepidermal Water Loss of Mice Skin

The effect of sesamin on UVB-induced erythema was detected by using a spectrocolorimeter (SCM-108, Laiko company, Tokyo, Japan), and the transepidermal water loss (TEWL) on the dorsal skin of hairless mice was measured in the 10th week by using a Tewameter TM 300 (Courage + Khazaka electronic GmbH, Cologne, Germany) as previously described [24].

#### 2.7.4. Immunohistological Analysis

The skin samples were incubated with primary antibodies for MMP-1, IL-6, NF-κB, and i-NOS. After being washed with PBS twice, the skin slides were incubated with the secondary antibody. The samples were examined under a microscope.

### 2.8. Statistical Analysis

Data are presented as mean ± standard deviation from at least triplicate independent experiments in vitro study. Statistically significant differences between the groups were determined using the Student’s *t*-test or ANOVA. *p* < 0.05 was considered to be significant.

## 3. Results

### 3.1. Sesamin Did Not Cause Cytotoxicity in Hs68 Cells

**Figure 1:**
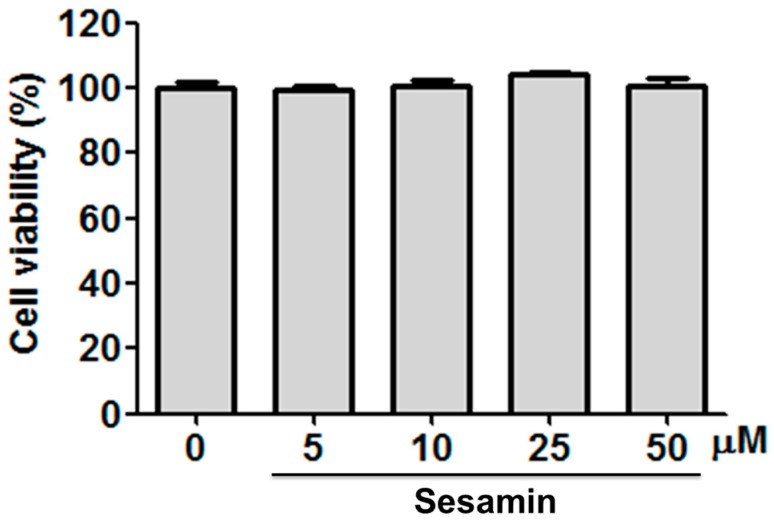
The cell viability (%) of sesamin on human skin fibroblasts and sesamin did not exhibit toxicity to the cells.

The cell viability of Hs68 cells treated with sesamin (5–50 μM) was assessed using the 3-(4,5-dimethylthiazol-2-yl)-2,5-diphenyltetrazolium bromide assay. The cell viability levels were over 95% of the control after the sesamin treatment, indicating that sesamin did not produce cytotoxic effects in Hs68 cells (Figure 1).

### 3.2. Sesamin Inhibited Intracellular ROS Formation in Hs68 Cells

**Figure 2:**
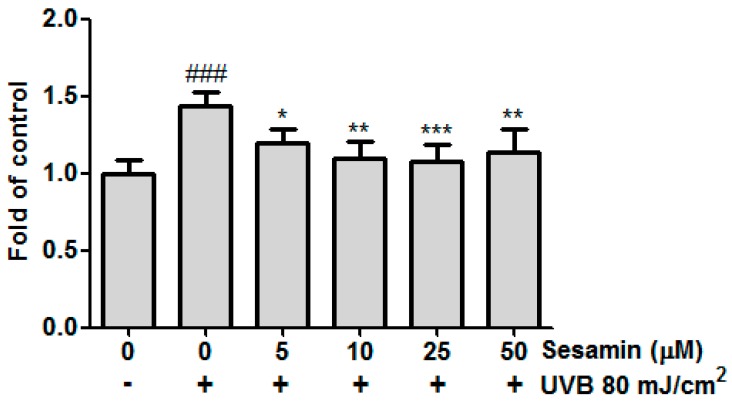
Sesamin inhibited the intracellular oxidative stress in Hs68 cells after ultraviolet (UVB) exposure. ### *p* < 0.001: Significant difference versus non-irradiation group. * *p* < 0.05; ** *p* < 0.01; *** *p* < 0.001: Significant difference versus non-treatment group.

ROS formation is a critical factor in intrinsic and extrinsic skin aging. The production of ROS in skin fibroblasts was detected through the DCFDA staining and the ROS were examined under an enzyme-linked immunosorbent assay reader. UVB irradiation significantly increased intracellular ROS generation by 1.4 ± 0.1-fold compared with the control group. However, ROS formation was significantly inhibited after treatment with sesamin at concentrations over 10 μM (Figure 2). These results indicate that sesamin can reduce UVB-induced intracellular ROS formation in Hs68 cells.

### 3.3. Effects and Mechanisms of Sesamin on Skin Photodamage

#### 3.3.1. Sesamin Inhibited UVB-Induced Overexpression of MMPs and Increased TIMP Expression

**Figure 3:**
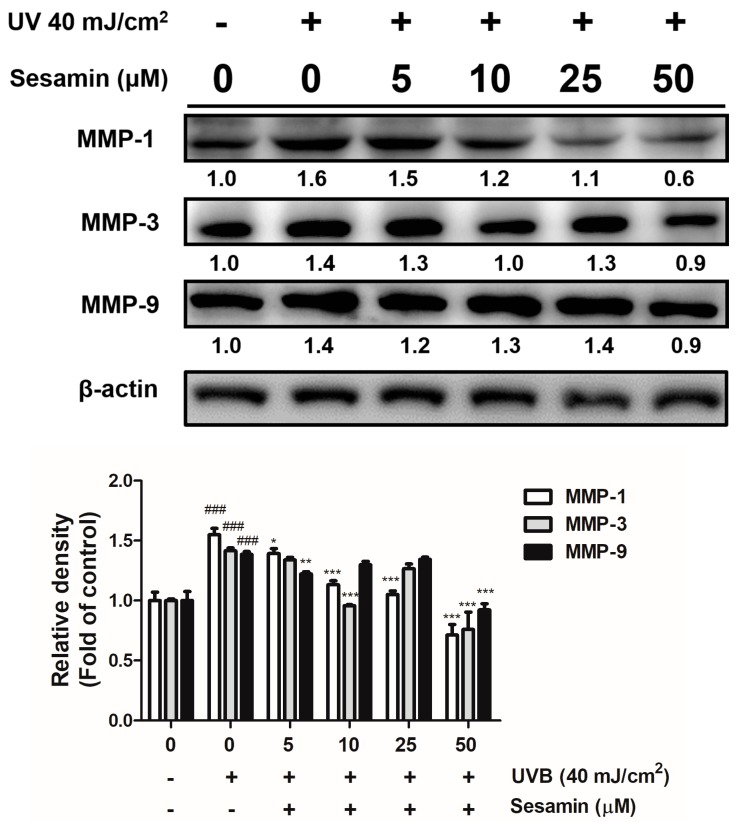
Sesamin inhibited the UVB induced matrix metalloproteinases (MMPs) expression in human skin fibroblasts. ###, *p* < 0.001: Significant difference versus non-irradiation group. * *p* < 0.05; ** *p* < 0.01; *** *p* < 0.001: Significant difference versus non-treatment group.

**Figure 4:**
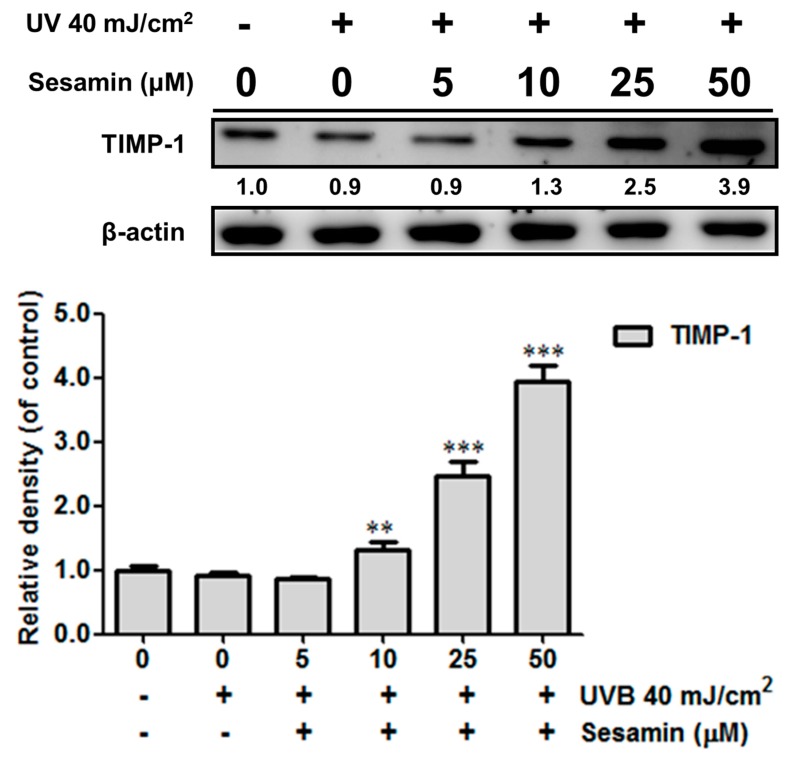
Sesamin reversed the UVB-inhibited tissue inhibitor of metalloproteina-1 (TIMP-1) expression in human skin fibroblasts. ** *p* < 0.01; *** *p* < 0.001: Significant difference versus non-treatment group.

UV irradiation resulted in the overexpression of MMP-1, -3, and -9 by 1.6-, 1.4-, and 1.4-fold compared with that of the control group; however, the pretreatment with 5–50 μM sesamin decreased MMP-1, -3, and -9 expressions in the Hs68 cells (Figure 3). Sesamin at doses over 5 μM significantly decreased the expression of MMP-1 by 1.5-fold compared with that of the control group, and that at a dose of 50 μM significantly reduced MMP-3 and MMP-9 expression by 0.9- and 0.9-fold compared with that of the control group (Figure 3). UVB inhibited TIMP-1 expression, which is a glycoprotein and natural inhibitor of MMPs (Figure 4). The sesamin treatment at 50 μM elevated the protein expression of TIMP-1 by 3.9-fold compared with that of the control group. These results signify that sesamin inhibited the expression of MMPs and upregulated the expression of TIMP-1 to protect the skin from UVB-irradiation-induced damage.

#### 3.3.2. Sesamin Inhibited UVB-Induced Overexpression of c-Jun/*p*-c-Jun

**Figure 5:**
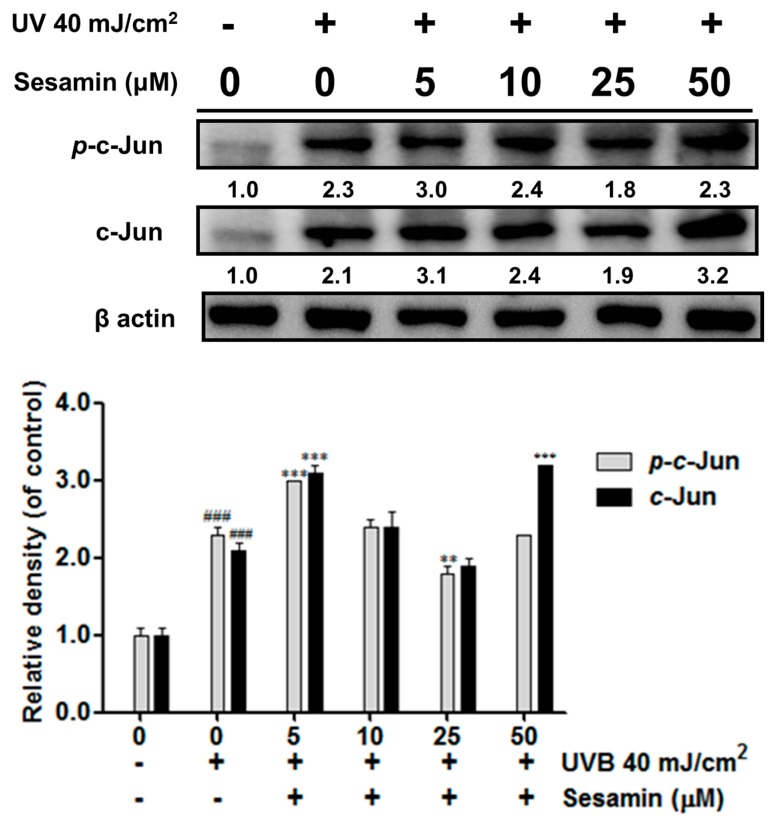
Sesamin on the ameliorated UVB induced AP-1 expression in human skin fibroblasts. ###, *p* < 0.001: Significant difference versus non-irradiation group. ** *p* < 0.01; *** *p* < 0.001: Significant difference versus non-treatment group.

UVB exposure upregulated the expression of *p*-c-Jun by 2.3-fold compared with the control group and that of c-Jun by 2.1-compared with the control. However, the treatment with sesamin at 25 μM significantly decreased the expression of c-Jun and *p*-c-Jun (Figure 5).

#### 3.3.3. Sesamin Inhibited the Upregulation of MAP Kinases Induced by UVB Irradiation

**Figure 6:**
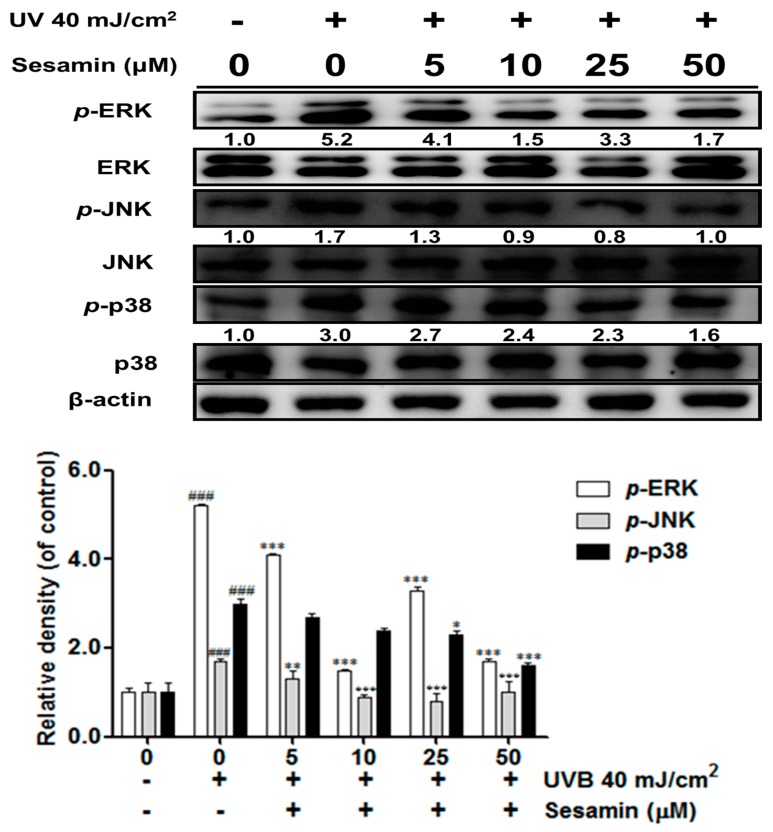
Sesamin inhibited on the UVB induced phosphorylation of mitogen-activated protein (MAP) kinases in human skin fibroblasts. ###, *p* < 0.001: Significant difference versus non-irradiation group. * *p* < 0.05; ** *p* < 0.01; *** *p* < 0.001: Significant difference versus non-treatment group. JNK: c-Jun N-terminal kinases.

UVB irradiation induced MAP kinases activation, which resulted in the upregulation of MMPs. The protein expression levels of *p*-ERK, *p*-JNK, and *p*-p38 were 5.2-, 1.7-, and 3.0-fold, respectively, compared with the control group after UVB irradiation (Figure 6); nevertheless, this effect was significantly inhibited after treatment with sesamin. Sesamin at 50 μM significantly reduced *p*-ERK and *p*-p38 expression and *p*-JNK expression at doses over 10 μM.

#### 3.3.4. Sesamin Modulated the Expressions of Smad3 and Smad7

**Figure 7:**
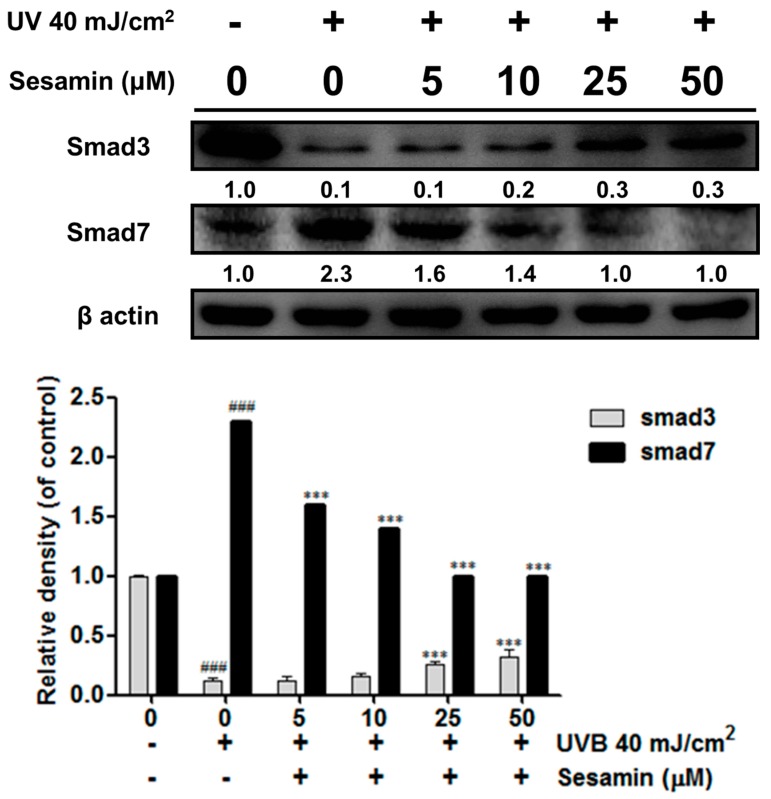
Sesamin modulated the UVB-mediated Smad 3 and Smad 7 expression in human skin fibroblasts. ### *p* < 0.001: Significant difference versus non-irradiation group. *** *p* < 0.001: Significant difference versus non-treatment group.

UVB irradiation reduced mothers against decapentaplegic homolog 3 (Smad3) expression and increased Smad7 expression in Hs68 cells (Figure 7). This may lead to the suppression of collagen biosynthesis. UVB exposure decreased Smad3 protein expression by 0.1-fold compared with that of the control group, whereas sesamin increased it by 0.3-fold compared with that of the control. In contrast to Smad3, Smad7 expression was increased by 2.3-fold relative to the control group after the UVB exposure and reduced by 1.6-fold compared with that of the control group after 5 μM of the sesamin treatment (Figure 7). In contrast to Smad7, Smad3 induced COA1A2 promotor activity, stimulating type I collagen expression [25]. These results indicate that sesamin promoted collagen synthesis by regulating Smad expression.

#### 3.3.5. Sesamin Attenuated UVB-Inhibited Total Collagen Biosynthesis

**Figure 8:**
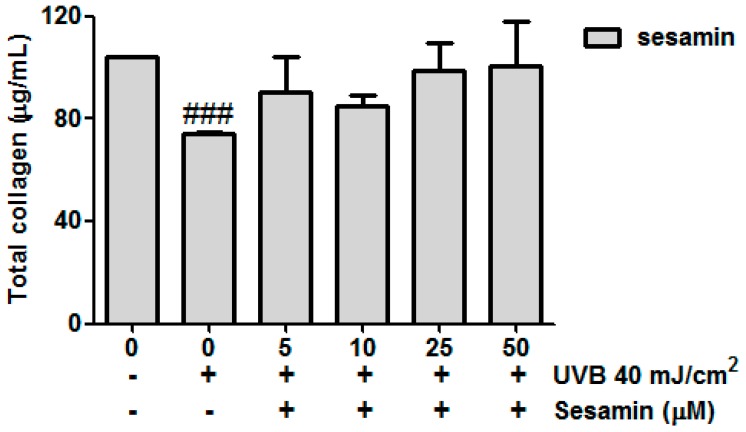
Effect of sesamin on the total collagen biosynthesis in human skin fibroblasts. ### *p* < 0.001: Significant difference versus non-irradiation group.

The total collagen production was 104.0 ± 0.0 μg/mL in the non-irradiation and non-treatment group and decreased to 74.0 ± 0.9 μg/mL after the UVB exposure (Figure 8). Sesamin increased collagen synthesis, and the sesamin treatment at 50 μM resulted in a collagen level of 108.0 ± 17.0 μg/mL (Figure 8). These results signify that sesamin could increase collagen synthesis by modulating Smad3/7 protein expression.

### 3.4. Anti-Inflammatory Effect of Sesamin

#### 3.4.1. Sesamin Ameliorated UVB-Induced Overexpression of i-NOS and COX-2

**Figure 9:**
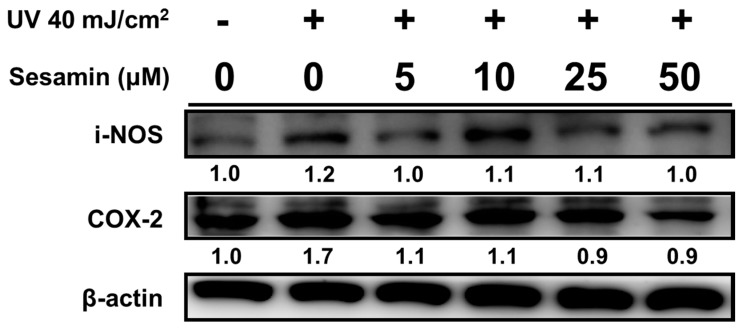

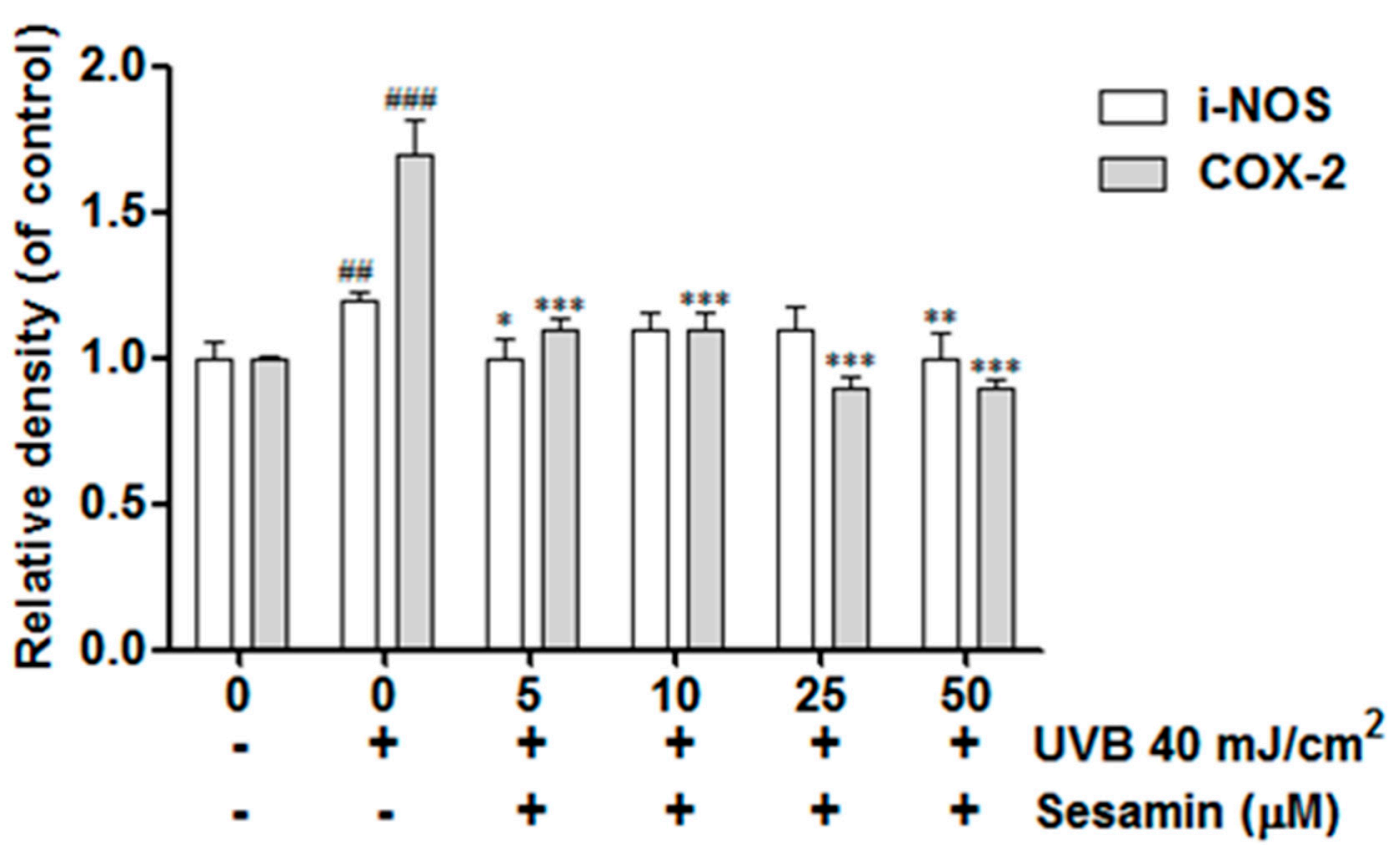
Sesamin inhibited the UVB induced inducible nitride oxide synthase (i-NOS) and cyclooxygenase (COX-2) in human skin fibroblasts. ## *p* < 0.01; ### *p* < 0.001: Significant difference versus non-irradiation group. * *p* < 0.05; ** *p* < 0.01; *** *p* < 0.001: Significant difference versus non-treatment group.

The expression levels of i-NOS and COX-2 were increased by 1.2- and 1.7-fold, respectively, relative to those of the control group after the UVB irradiation (Figure 9). After the sesamin treatment at 5 μM, the i-NOS and COX-2 protein expression levels were significantly reduced. Moreover, the sesamin treatment at 5 μM reduced i-NOS expression in fibroblasts by 1.0-fold compared with that of the control group, and the treatment at 25 μM reduced COX-2 expression by 0.9-fold.

#### 3.4.2. Sesamin Reduced COX-2 Expression by Inhibiting MAP Kinase Expression

**Figure 10:**
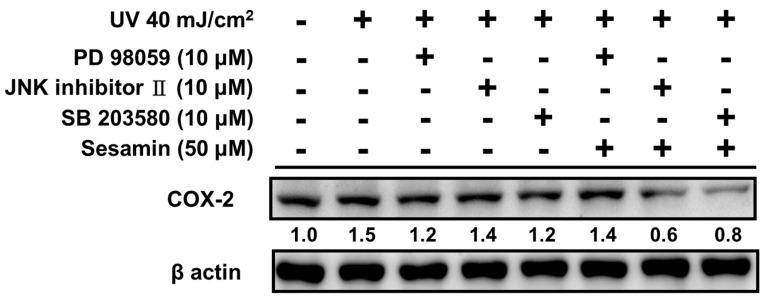

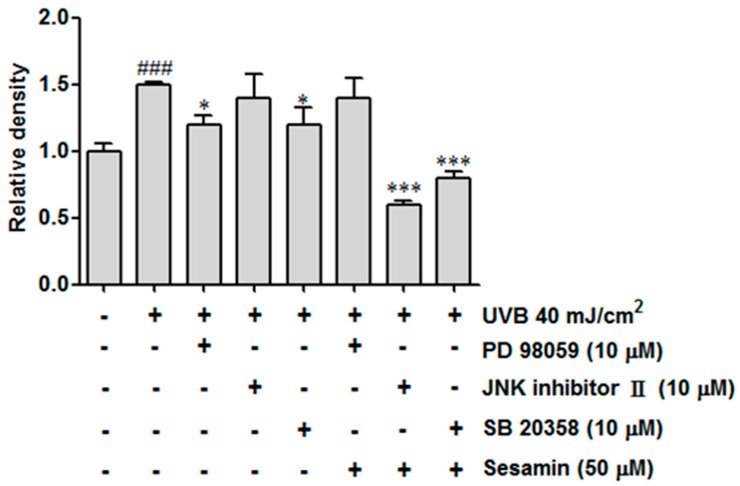
Effects of the MAP kinase inhibitor and sesamin on the UVB induced COX-2 expression in human skin fibroblasts. ### *p* < 0.001: Significant difference versus non-irradiation group. * *p* < 0.05; *** *p* < 0.001: Significant difference versus non-treatment group.

The phosphorylation of MAP kinases can activate COX-2 expression, causing inflammation. COX-2 expression was decreased after the treatment with 10 μM PD98059 (ERK inhibitor), the JNK inhibitor II, and SB203580 (p38 inhibitor), as presented in Figure 10. The cotreatment with sesamin and MAP kinase inhibitors further reduced the expression of COX-2. Sesamin inhibited the MAP kinase upregulation after the UVB exposure, leading to the inhibition of COX-2 expression.

#### 3.4.3. Sesamin Inhibited UVB-Induced NF-κB Activation

**Figure 11:**
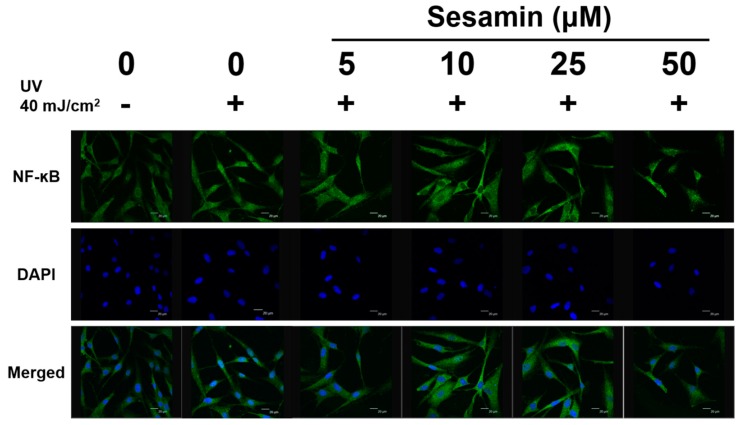
Effect of sesamin on the UVB-induced activation of nuclear factor κB p65 in human skin fibroblasts.

UVB irradiation triggered NF-κB activation, which caused movement from the cytoplasm to the nucleus of Hs68 cells. After the treatment with sesamin, the translocation of NF-κB was diminished (Figure 11). NF-κB activation is an indicator of inflammation, and the results suggest that sesamin can inhibit the UVB-induced inflammation in skin cells.

### 3.5. Sesamin Protected Mouse Skin from UVB-Irradiation-Induced Damage

#### 3.5.1. Sesamin Reduced UVB-Induced Skin Erythema and Damage

**Figure 12:**
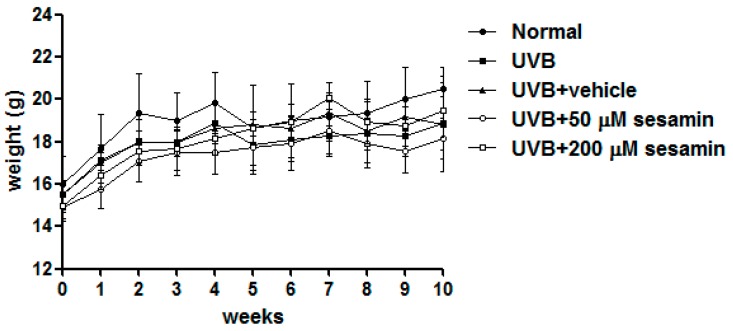
Body weight of hairless mice during 10 weeks.

**Figure 13:**
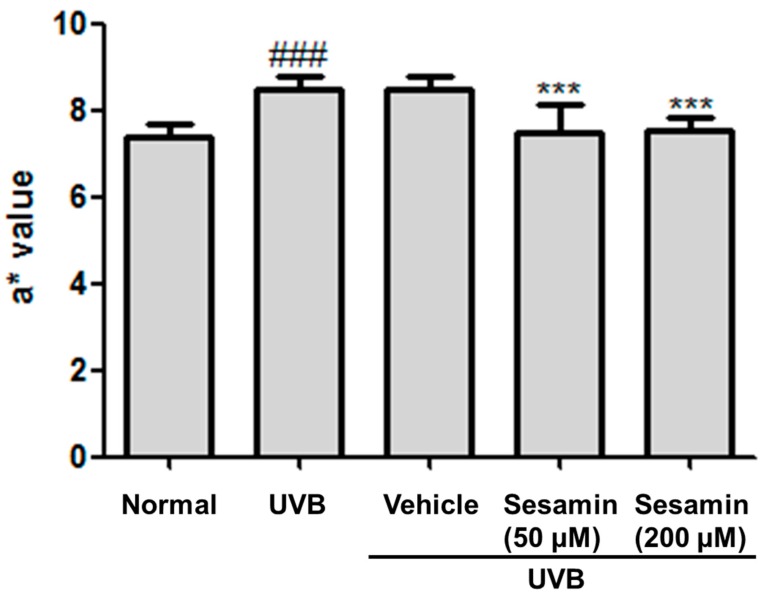
Effect of sesamin on the a* value in chronic UVB-irradiation hairless mice at the 10th week. Significant difference versus normal group: ### *p* < 0.001. Significant difference versus UVB group: *** *p* < 0.001.

After 10 weeks of UV irradiation and treatment with sesamin, no significant differences in body weight were found between the groups (Figure 12). The a* value is a parameter of the degree of erythema and inflammation. The a* values significantly increased in the fourth week, meaning the UVB exposure caused the skin erythema and inflammation (Figure 13). After 10 weeks, the a* values observed for the UVB-irradiated and 200-μM-sesamin-treated mice were similar to those observed for the normal mice, indicating that the sesamin treatment decreased erythema. These results from the animal study were consistent with those observed for the sesamin treatment on the UVB-induced inflammation in Hs68 cells.

**Figure 14:**
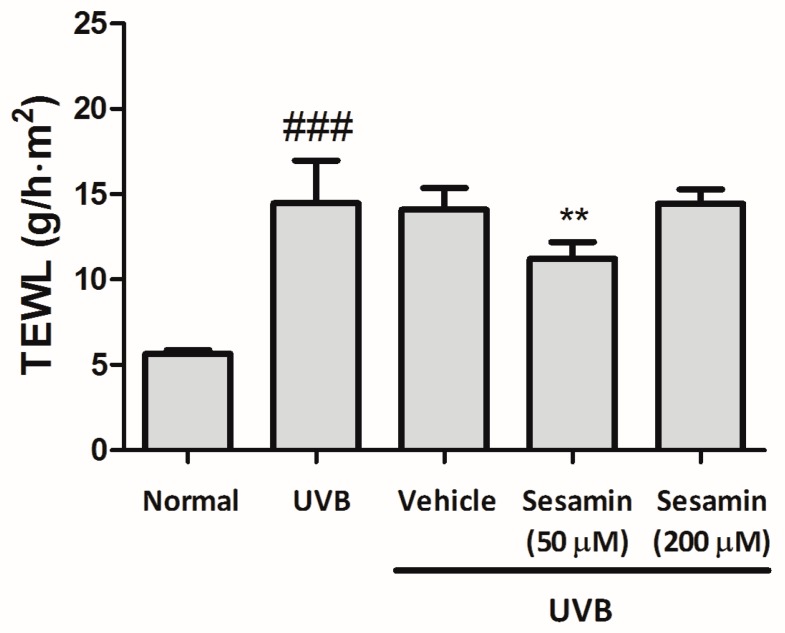
Effect of sesamin on the transepidermal water loss (TEWL) in chronic UVB-irradiation hairless mice at the 10th week. Significant difference versus normal group: ### *p* < 0.001. Significant difference versus UVB group: ** *p* < 0.01.

Skin damage may increase the transepidermal water loss (TEWL). In this study, TEWL was increased (14.5 ± 2.5 g/h·m^2^) after the UVB exposure for 10 weeks (Figure 14). However, with the topical application of sesamin to the hairless mice for 10 weeks, TEWL was significantly reduced to 11.2 ± 1.0 g/h·m^2^ (Figure 14). These results signify that sesamin was not toxic to the skin; additionally, it protected the skin from UV damage.

#### 3.5.2. Sesamin Reduced UVB-Induced Wrinkle Formation

**Figure 15:**
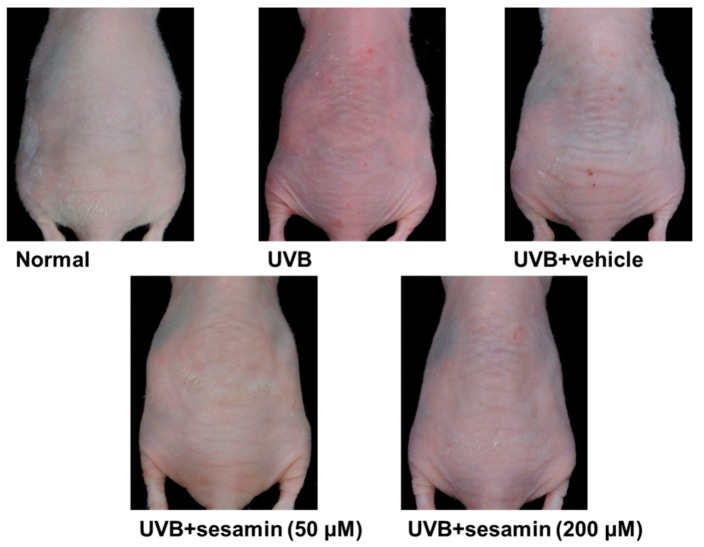
Photographs show skin wrinkles induced by the chronic UVB irradiation and the effect of topically applied sesamin.

Wrinkle formation was examined macroscopically in the dorsal region following the initiation of UVB irradiation, and the images were captured by a camera (Figure 15). Table 1 presents the scores of wrinkle formation assessed from the mouse images according to the grading scale used in previous research [23,26]. Topically applying sesamin at 50 and 200 μM ameliorated the wrinkle production (Figure 15). The wrinkle score was 4.5 ± 1.9 in the UVB-irradiated group and significantly decreased to 1.3 ± 1.6 and 1.3 ± 1.0 in the 50- and 200-μM-sesamin-treated groups, respectively (Table 1). These results indicate that the topical application of sesamin for 10 weeks significantly reduced wrinkle formation induced by the chronic UVB exposure.

#### 3.5.3. Sesamin Reduced UVB-Induced Epidermal Hyperplasia and Restored Collagen Content

**Figure 16:**
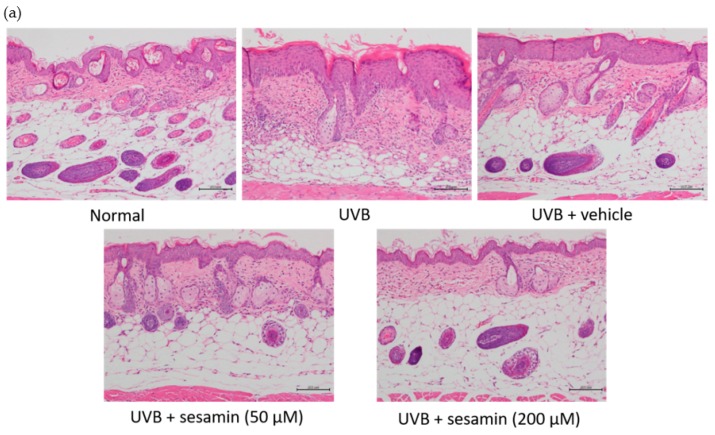

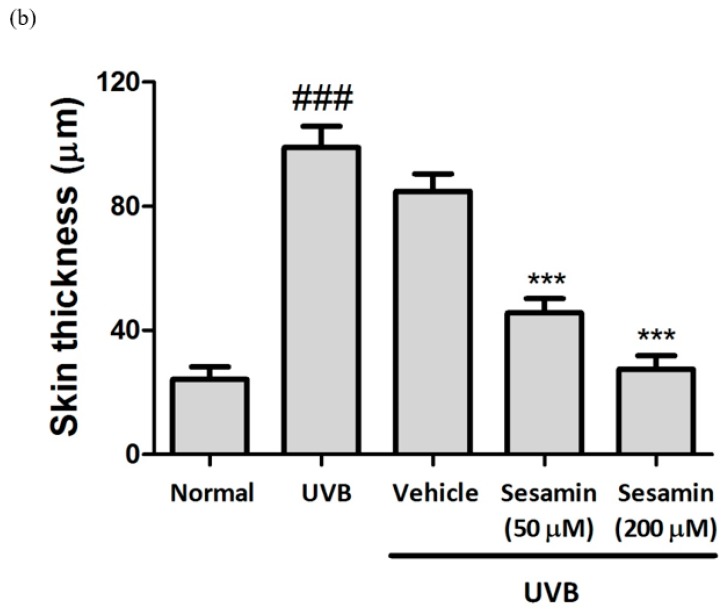
Effect of sesamin on skin thickness in chronic UVB-irradiation hairless mice. (**a**) Light micrographs of histological sections stained with hematoxylin and eosin (H&E); and (**b**) effect of sesamin on skin thickness in chronic UVB-irradiation hairless mice at the 10th week. Significant difference versus normal group: ### *p* < 0.001. Significant difference versus UVB group: *** *p* < 0.001.

**Figure 17:**
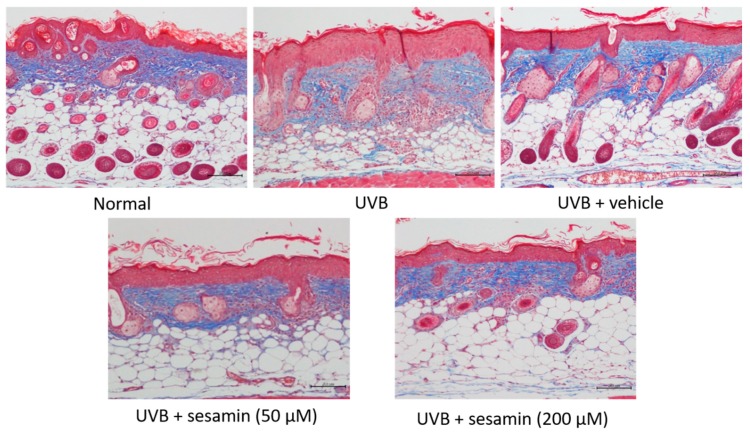
Effect of sesamin on the skin collagen content in chronic UVB-irradiation hairless mice. Light micrographs of histological sections stained with Masson’s trichrome in hairless mice. Collagen fibers were stained in blue.

A histological examination was conducted to assess the effect of sesamin on the thickness and collagen density of the hairless mouse skin after chronic exposure to UVB irradiation for 10 weeks. UVB irradiation significantly increased the skin thickness of the mice, whereas the topical application of sesamin reduced the thickness of the skin (Figure 16a,b). The epidermal thickness was 24.2 ± 4.1 μm in the control group and 99.0 ± 6.8 μm in the UVB-irradiated group. The epidermal thickness was 45.6 ± 4.6 μm in the 50-μM-sesamin-treated group and 27.5 ± 4.4 μm in the 200-μM-sesamin-treated group (Figure 16b). Masson’s trichrome staining revealed that the collagen content in the dermis in the UVB-irradiated group was decreased compared with that in the control group; however, the sesamin treatment increased the collagen content in the mouse dermis (Figure 17). These results suggest that sesamin significantly ameliorated UVB-irradiation-induced skin hyperplasia and elevated the collagen content.

#### 3.5.4. Sesamin Inhibited Photodamage-Related Protein Levels in UVB-Irradiated Mouse Skin

**Figure 18:**
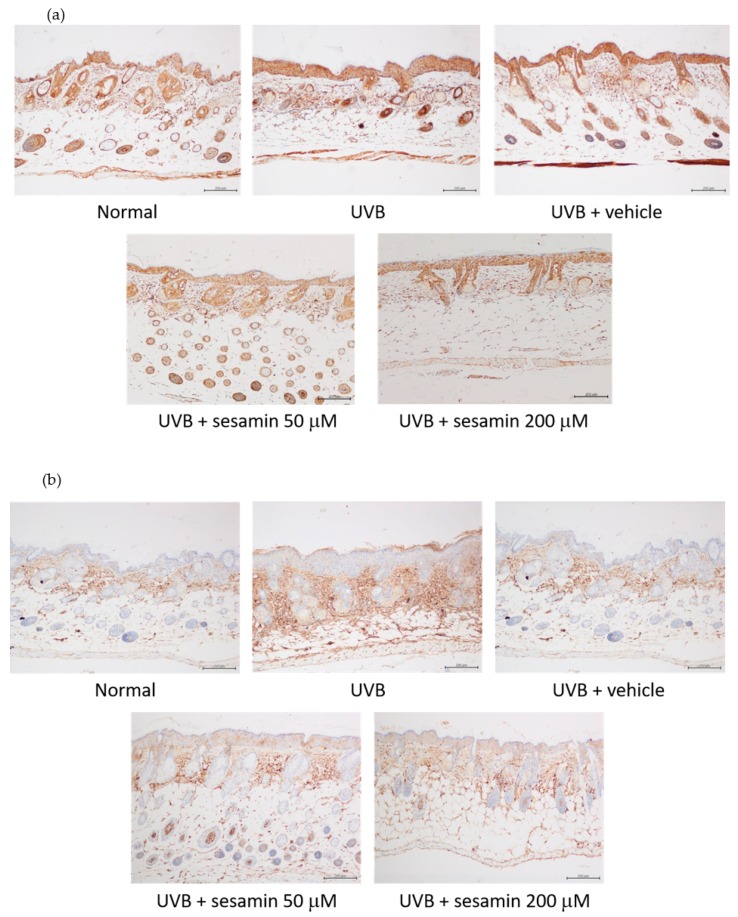

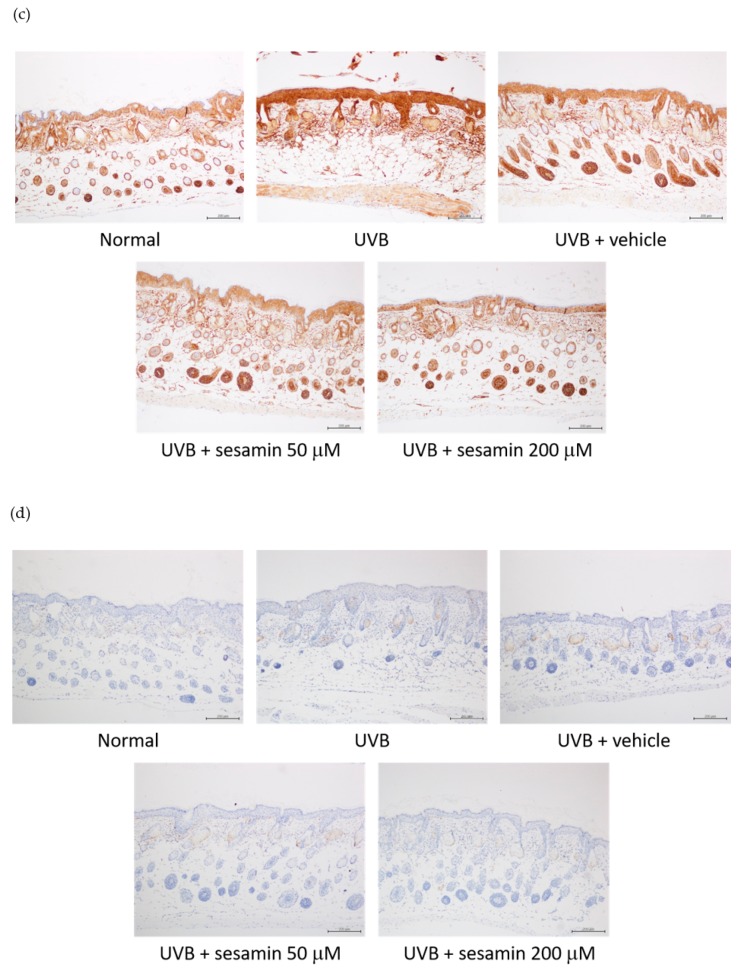
Immunohistological staining of skin slices for (**a**) MMP-1, (**b**) IL-6, (**c**) NF-κB, and (**d**) i-NOS in the hairless mouse skin after chronic UVB exposure and sesamin treatment.

MMP-1, IL-6, NF-κB, and i-NOS expression increased in the dermis of hairless mice after exposure to UVB irradiation for 10 weeks; however, the sesamin treatment reduced the expression of these four proteins or mediated overexpression (Figure 18a–d). These results indicate that UVB-induced MMP-1 overexpression caused collagen degradation in the skin and that sesamin reversed this effect. Additionally, UVB induced inflammation in the skin, causing the increase in IL-6, NF-κB, and i-NOS protein levels, whereas the sesamin treatment inhibited these effects. The results observed for mice were consistent with those observed for human skin fibroblasts.

## 4. Discussion

Oxidative stress is one of the major factors for aging, and the biochemical reaction associated with normal metabolic processes often produces free radicals and ROS [27,28]. UV irradiation promotes ROS generation and triggers aging-related signal transduction, resulting in skin sagging, rough skin, hyperpigmentation, and skin cancers [29]. Many reports have demonstrated that natural products with antioxidants exhibit anti-inflammatory and antiphotoaging activities [30,31,32]. Sesame and its major active component, sesamin, were reported to exhibit free-radical-scavenging and antioxidative activities [33,34]. In the present study, sesamin inhibited UVB-induced ROS formation in skin fibroblasts; therefore, it may be used as a photoprotective agent.

Long-term exposure to UV causes collagen degradation in the dermis, leading to photodamage. UV exposure induces the skin damage by promoting collagen degradation or collagen synthesis inhibition through the regulation of the transforming growth factor (TGF)-β/Smad pathway. MMP-1 degrades the collagen fiber bundles, and, subsequently, MMP-3 promotes the activity of MMP-1, further degrading collagen to fragments. MMP-9 can further degrade the MMP-1 cleaved collagen [35,36]. The C-terminal domain and N-terminal domain of TIMPs can conjugate with the active site of MMPs and inhibit them, resulting in the inhibition of collagen damage [37]. The results of this study show that the sesamin treatment could block UVB-induced collagen degradation by inhibiting MMP-1, -3, and -9 expressions in human skin fibroblasts. Furthermore, sesamin elevated the expression of TIMP-1. In the animal study, results demonstrate that sesamin exhibited potent antioxidant activity and ameliorated wrinkle formation induced by chronic UVB exposure.

UV irradiation increased intracellular oxidative stress and induced the phosphorylation of MAP kinases, resulting in the transcription factor AP-1 translocating into the nucleus to trigger secretion of MMPs [38]. Sesamin inhibited UVB-induced AP-1 and the phosphorylation of ERK, JNK, and p38 proteins. Moreover, UV irradiation inhibited collagen biosynthesis in fibroblasts. TGF-β is a multifunction regulator and modulates the growth, differentiation, apoptosis, migration, adhesion, and immune response of cells [39,40]. The TGF-β pathway is the major pathway of type Ⅰ procollagen synthesis. TGF-β activates the combination of downstream proteins, Smad2, and Smad3, which bind with Smad4 to form a complex [41]. The Smad complex enters the nucleus to synthesize the type Ⅰ procollagen. UV irradiation activates Smad7 to inhibit the TGF-β receptor and block signal transduction, leading to the inhibition of collagen synthesis [42]. Our results suggest that sesamin inhibited Smad7 overexpression and increased Smad3 expression to increase the content of total collagen.

Overexposure to UV irradiation triggered the activation of the MAP kinase pathway and of NF-ĸB, resulting in COX-2 and i-NOS protein expression and then causing skin erythema and inflammation. The results of this study indicate that UVB upregulated COX-2 and i-NOS protein expression and NF-ĸB translocation in human skin fibroblasts, whereas sesamin inhibited these effects. The results signify that treatment with sesamin ameliorated UVB-induced skin inflammation. A previous study reported that sesamin inhibited inflammation of neurons in rats with intracerebral hemorrhage by suppressing ERK and p38 activation [43]. Sesamin protected neurons from lipopolysaccharides damage by inhibiting the p38 MAP kinase pathway and NF-κB activation [15]. Additionally, cotreatment with the JNK or p38 inhibitor and sesamin significantly reduced the protein expression of COX-2; thus, sesamin may inhibit COX-2 expression through the JNK and p38 pathways, resulting in anti-inflammation. UVB can activate the p38 pathway to induce skin inflammation and can even lead to cancer [44,45,46].

Chronic exposure to UV causes photodamaging of the skin and induces various skin disorders, including sunburn, dryness, wrinkles, and skin cancer [47]. UV light also can induce oxidative stress and inflammation of the skin. After UVB exposure for two and four weeks, the a* value was increased and then significantly increased at 10 weeks, indicating inflammation of the mouse skin. The sesamin treatment reversed UVB-induced skin erythema and inflammation. The skin is a barrier that protects the body from deleterious factors in the environment and prevents water loss, in addition to maintaining the homeostasis of water and electrolytes [48,49]. TEWL may be an index for the barrier function of the skin. TEWL increases when the stratum corneum is damaged. In this study, long-term UVB exposure caused the TEWL to increase; however, TEWL was not significantly changed after the sesamin treatment for 10 weeks, indicating that sesamin did not cause skin toxicity.

UV exposure induces degradation of ECM in the dermis structural and compositional remodeling in dermis, resulting in the wrinkle and sagging of the skin [50]. In the evaluation of wrinkle formation, chronic exposure to cumulative UVB for 10 weeks caused significant wrinkle formation on mice dorsal skin. However, the topical application of sesamin reduced UVB-induced wrinkles on the skin. In addition, UVB-induced epidermal thickness decreased significantly after the sesamin treatment. After UVB exposure, brown granulates were present in the epidermis, indicating immune cell infiltration in the skin. Sesamin reduced the infiltration of leukocytes in the skin. The results of this study indicate that sesamin inhibited UVB-induced skin inflammation. UVB decreased collagen in the dermis, and the vehicle slightly increased the content of collagen. Sesamin significantly increased the content and density of collagen in the dermis, which may reduce UV-induced skin damage. UV irradiation caused hyperplasia of the epidermis, reduction of collagen content, and denaturation of elastin, leading to skin photoaging. Sesamin ameliorated skin hyperplasia and collagen degradation.

## 5. Conclusions

In summary, sesamin elevated TIMP-1 protein expression and suppressed MAP kinase phosphorylation, resulting in the downregulation of MMP expression. Sesamin also inhibited COX-2 and i-NOS protein expression through MAP kinase inhibition to reduce NF-ĸB activation, resulting in anti-inflammation induced by the UVB exposure. Sesamin decreased UVB-induced skin erythema to ameliorate photoinflammation and reduced epidermal thickness and collagen content in the dermis to protect the skin from wrinkle formation and photodamage.

## Figures and Tables

**Table 1:** Effect of sesamin on skin wrinkles induced by the UVB irradiation in hairless mice.

Group	Wrinkle Score (10th Week)
Normal mice	0.7 ± 1.2 ^a^
UVB-irradiated mice	4.5 ± 1.9 ^b^
UVB-irradiated mice + vehicle	5.3 ± 1.2 ^b^
UVB-irradiated mice + sesamin (50 μM)	1.3 ± 1.6 ^a,c^
UVB-irradiated mice + sesamin (200 μM)	1.3 ± 1.0 ^a,c^

Values not followed by a common letter are significantly different (*p* < 0.05).
